# Trend of anticoagulant therapy in elderly patients with atrial fibrillation considering risks of cerebral infarction and bleeding

**DOI:** 10.1038/s41598-022-26741-7

**Published:** 2023-01-05

**Authors:** Noriko Tsuji, Yoshimitsu Takahashi, Michi Sakai, Shosuke Ohtera, Junji Kaneyama, Kosai Cho, Genta Kato, Shigeru Ohtsuru, Takeo Nakayama

**Affiliations:** 1 Department of Health Informatics, Graduate School of Medicine and Public Health, Kyoto University, Kyoto, Japan; 2 Comprehensive Unit for Health Economic Evidence Review and Decision Support, Ritsumeikan University, Kyoto, Japan; 3 Center for Outcomes Research and Economic Evaluation for Health, National Institute of Public Health, Wako, Japan; 4 Second Department of Cardiology, Akita Cerebrospinal and Cardiovascular Center, Akita, Japan; 5 Department of Primary Care and Emergency Medicine, Kyoto University Hospital, Kyoto, Japan; 6 Solutions Center for Health Insurance Claims Integrated Clinical Education Center, Kyoto University Hospital, Kyoto, Japan

**Keywords:** Thrombosis, Atrial fibrillation

## Abstract

The introduction of direct oral anticoagulants (DOACs) has greatly changed the use of anticoagulant therapy in patients with non-valvular atrial fibrillation (Af). Therefore, this study aimed to examine changes in the proportions of oral anticoagulant prescriptions in patients with non-valvular Af aged ≥ 65 years, taking into consideration the risk of cerebral infarction and bleeding. Anticoagulant prescriptions in outpatients aged ≥ 65 years with Af were temporally analyzed using the nationwide claims database in Japan. Trends in anticoagulant prescriptions were examined according to cerebral infarction and bleeding risk. The proportion of anticoagulant prescriptions for 12,076 Af patients increased from 41% in 2011 to 56% in 2015. An increase in DOAC prescriptions was accompanied by an increase in the proportion of anticoagulant prescriptions in each group according to the CHA2DS2-VASc and HAS-BLED scores. The proportion of anticoagulant prescriptions for patients with a high risk of developing cerebral infarction and bleeding showed a marked increase. Trends in anticoagulant prescriptions in Af patient with a CHA_2_DS_2_-VASc score ≥ 2 and HAS-BLED scores ≥ 3 showed a marked increase in DOAC prescriptions. The widespread use of DOACs greatly changes the profile the prescription of anticoagulant therapy in patients with Af.

## Introduction

Atrial fibrillation (Af) is a common arrhythmia in the elderly. Af is associated with the development of cardiogenic cerebral infarction^1^, with serious outcomes including physical dysfunction and death^2^.

In order to prevent the development of cardiogenic cerebral infarction, Af patients are conventionally prescribed warfarin potassium (vitamin K antagonist: VKA), an anticoagulant agent. With the release of dabigatran in 2011, prescriptions for direct oral anticoagulants (DOACs) have increased rapidly^3–9^. There have been several Japanese database studies reporting increase of DOACs prescriptions^10–13^. One of them used the prefecture-level the National Health Insurance database ^10^ and three of them used the Japan Medical Data Center (JMDC) database^11–13^, which did not include the patients 75 years or older. The recent two studies that also included old patients, one used the Japanese Diagnosis Procedure Combination (DPC) database including claim data of acute care hospitals^14^ and the other used the National Database sampling dataset^15^, added findings on prescription patterns of DOACs. However, there have been no papers reporting the trend of prescriptions of DOACs stratified by risks of cerebral infarction and bleeding at the nation-level including old patients. While recent guidelines around the world recommend prescribing DOACs^16–19^, it remains unclear how indications and risk of bleeding, a serious side effect of anticoagulants, are considered when these drugs are prescribed. Accordingly, the present study aimed to reveal the state of anticoagulant prescriptions in Japanese elderly Af patients aged ≥ 65 years, considering the risk of cerebral infarction and bleeding. To this end, trends in anticoagulant prescriptions were examined using the National Database of Health Insurance Claims and Specific Health Checkups of Japan (NDB), a highly exhaustive claims database that stores claims information collected from over 95% of the Japanese population, from infants to elderly individuals^20–24^.

## Methods

### Study design and database used

This cross-sectional study used the NDB sampling dataset which sampled from whole the claims database of NDB to raise convenience of the NDB utilization^20^. In order to examine annual changes of OAC prescriptions without following up patients, we conducted this study and considered that the sampling dataset was suitable for this study. Of the entire dataset available at the time of the study (including data from inpatients, outpatients, and pharmacy claims, as well as DPC data; note that pharmacy claims include data for November, which are linked to outpatient data), we applied to and obtained approval from the Ministry of Health, Labour and Welfare for the use of patients claims data from October of each year from 2011 to 2015. As it was thought that the seasonal change was not related to examine annual changes of the OAC prescriptions very much, we decided to examine annual changes using patients claims data from October of each year from 2011 to 2015.

This NDB sampling dataset comprises data (10% each for inpatients and DPC data, and 1% each for outpatients and pharmacy claims data) extracted from a highly exhaustive claims database^21–25^ that stores claims information collected from over 95% of the Japanese population, including those aged 75 or older, as opposed to other studies^10–12^.

In this study, we examined outpatients and pharmacy claims data of patients coming to hospital in October each year and find out the trend to everyday OAC prescriptions without limiting on to new patients. This database includes data on patient age and sex, ambulatory services and diagnoses, medical treatment, and aid, as well as drug dispensation. All diagnoses in the database were coded according to the 10th revision of the International Classification of Diseases (ICD-10). Pharmacy claims include various information on outpatient prescriptions dispensed from Japan pharmacies, such as dispensing/prescription dates and the number of tablets dispensed. Prescription handouts were coded according to the Anatomical Therapeutic Chemical Classification System.

### Subjects

We included patients aged ≥ 65 years only with the definitive disease code corresponding to the ICD-10 code (I48: atrial fibrillation) in their medical claims and excluded those with suspicious disease codes^25^. Furthermore, we excluded patients with a disease code for valvular Af or valvular disease recorded in their medical claims (i.e., those with an ICD-10 code I05, I06, I34, or I35), those with venous thromboembolism (I802 or I269), and those who underwent artificial joint replacement (total hip arthroplasty, knee arthroplasty). The extracted non-valvular Af patients were included in this study (hereafter referred to as “Af patients”). Oral anticoagulants (OACs) were defined as VKA and four DOACs (dabigatran, rivaroxaban, apixaban, and edoxaban), coded in pharmacy claims data as 3332-D01280, 3339-D07082, D07086, D03213, and D09546, according to the ATC classification.

Patients who had been prescribed an antiplatelet agent (AP) were extracted, and those who had been prescribed both anticoagulants and AP were considered candidates for anticoagulant therapy. For drug codes, medical claim codes corresponding to the ATC classification numbers were used^25^. The list of drug and disease codes used for subject extraction is shown in Supplementary Table S1.

### Evaluation indices

The primary evaluation indices were proportions of Af patients who were prescribed VKA, DOACs, and AP in each October from 2011 to 2015, as well as those who were not prescribed any of the above-mentioned drugs, among all Af patients.

### Measurement item

Subject characteristics included age, sex, comorbidities (cerebral infarction, myocardial infarction, ischemic heart disease, peripheral artery disease, heart failure, chronic kidney disease, liver disease, hemorrhagic disease, alcoholic disease, hypertension, and diabetes mellitus), and concomitant drugs (non-steroidal anti-inflammatory drugs [NSAIDs]). Each disease was extracted using disease codes recorded in medical claims^25^ corresponding to ICD-10 codes. Patients with hypertension (defined as those who were prescribed antihypertensive drugs) and patients with diabetes (defined as those who were prescribed anti-diabetic drugs) were extracted and included as subjects. Patients with renal dysfunction, defined as those with a treatment code corresponding to dialysis recorded in their medical claims^25^, were also included. The list of drug and disease codes used for subject extraction is shown in Supplementary Table S1.

### CHA_2_DS_2_-VASc and HAS-BLED scores

The CHA_2_DS_2_-VASc score was used to assess the risk of developing cerebral infarction, and a total score was obtained by adding 1 point each for the presence of heart failure, hypertension, diabetes mellitus, and vascular disease (history of myocardial infarction, peripheral arterial disease, aortic plaques), female sex, and age 65–74 years, and 2 points each for age ≥ 75 years and prior cerebral infarction/TIA^26^. The HAS-BLED score was used to assess the risk of bleeding during anticoagulant therapy, and a total score was obtained by adding 1 point each for hypertension, prior history of cerebral infarction/TIA, kidney dysfunction (dialysis, kidney transplant), liver dysfunction (cirrhosis), prior bleeding or predisposition to bleeding, age ≥ 65 years, use of AP and NSAIDs, alcoholism, and unstable INR^27^. Since INR values were not available, the highest HAS-BLED score was 8 points.

### Analyses

Data were analyzed using descriptive statistics, followed by the Cochrane Armitage test to examine changes over time in prescriptions for each drug. The entire Af patient population was divided into four groups: two groups according to the CHA_2_DS_2_-VASc score (< 2 or ≥ 2) and two groups according to the HAS-BLED score (< 3, ≥ 3), based on data from to 2011–2015. The numbers of VKA and DOAC prescriptions and proportions of prescriptions for each of these drugs among all Af patients were calculated for each group. The first of the four groups comprised patients with “low thrombus risk and low bleeding risk,” for whom anticoagulant therapy is only considered an option (Group 1); the second group comprised of patients with “low risk of developing cerebral infarction and high risk of bleeding,” for whom caution is required when prescribing anticoagulant therapy (Group 2); the third group was made up of patients with “high risk of developing cerebral infarction and low risk of bleeding,” for whom anticoagulant therapy is recommended (Group 3); and the fourth group comprised patients with “both high risk of developing cerebral infarction and high risk of bleeding,” for whom clinical judgment is difficult due to opposing risks and benefits of anticoagulant therapy for non-valvular Af patients (Group 4).

We extracted study population using PostgreSQL 9.3.25^28^. Statistical analyses were performed using R (version 3.3.2) (Copyright (C) 2016 The R Foundation for Statistical Computing), with a two-tailed test at a significance level of 5%^29^. The graph and the flowchart were prepared using Adobe Illustrator CC2019^30^.

### Ethical considerations

This study complied with the guidelines of the Ministry of Health, Labour and Welfare regarding the provision of health insurance claims information and special health check-up information (revised April 2015)^23^ and was approved by the Ethics Committee of Kyoto University Faculty of Medicine (approval number: R1013).

## Results

### Subject extraction

The same subject extraction process was followed over a 5-year period. The representative results from 2015 are described below.

A total of 14,363 Af patients aged ≥ 65 years were extracted from the 2015 outpatient claims data. Of these, 12,076 Af patients were subject to analysis after excluding 2049 patients with valvular Af or valvular disease, 173 patients with venous thromboembolism, and 65 patients who were prescribed two or more anticoagulants. The characteristics of the patients are summarized in Table 1. Mean age (SD) was 78.8 (7.7) years, mean CHADS_2_ score (SD) was 2.5 (1.4), mean CHA_2_DS_2_-VASc score (SD) was 4.1 (1.5), and mean HAS-BLED score (SD) was 2.6 (1.1).

**Table 1:** Characteristics of atrial fibrillation (Af) patients aged ≥ 65 years (2015).

Variable	All		VKA		DOAC		None	
	(n = 12,076)		(n = 2991)		(n = 3770)		(n = 5315)	
Age (years) mean (SD)	78.8	(7.7)	79.1	(7.6)	77.9	(7.3)	79.2	(8.1)
Female n (%)	2724	(41)	1184	(40)	1559	(41)	2447	(46)
CHADS_2_ score mean (SD)	2.5	(1.4)	3.0	(1.3)	2.7	(1.3)	2.1	(1.3)
CHA_2_DS_2_-VASc score mean (SD)	4.1	(1.5)	4.5	(1.4)	4.2	(1.5)	3.7	(1.5)
HAS-BLED score mean (SD)	2.6	(1.1)	2.9	(1.0)	2.7	(1.0)	2.3	(1.1)
**Comorbidities n (%)**								
Cerebral infarction/TIA	2995	(25)	832	(28)	977	(26)	1164	(22)
Heart failure	6411	(53)	1931	(65)	1997	(53)	2447	(46)
Vascular disease	1609	(13)	431	(14)	461	(12)	709	(13)
Chronic kidney disease	1342	(11)	422	(14)	292	(8)	622	(12)
Liver damage	2403	(20)	599	(20)	711	(19)	1079	(20)
Bleeding	2727	(23)	773	(26)	760	(20)	1183	(22)
Alcoholism	147	(1.2)	40	(1.3)	47	(1.2)	59	(1.1)
Hypertension	7910	(65)	2583	(86)	3051	(81)	2226	(42)
Diabetes mellitus	1723	(14)	545	(18)	702	(19)	467	(9)
Dialysis	142	(1.2)	46	(1.5)	1	(0.0)	95	(1.8)
**Concomitant drugs n (%)**								
Antiplatelet drugs	702	(5.8)	174	(5.8)	195	(5.2)	327	(6.2)
NSAIDs	2005	(17)	584	(18)	679	(18)	762	(14)

*CHADS_*2*_* score* heart failure, hypertension, age ≥ 75 years, diabetes mellitus, prior cerebral infarction, or TIA(doubled).

^†^*CHA*_*2*_*DS*_*2*_*-VASc score* heart failure, hypertension, age ≥ 75 years (doubled), diabetes mellitus, prior cerebral infarction or TIA (doubled), vascular disease, age 65–74 years, female sex.

^‡^*HAS-BLED score* hypertension, renal and hepatic dysfunction, stroke, bleeding, age ≥ 65 years, concomitant antiplatelet drugs and NSAIDs, alcoholism.

^§^*TIA* transient ischemic attack.

^II^*NSAIDs* non-steroidal anti-inflammatory drugs.

**Figure 1:**
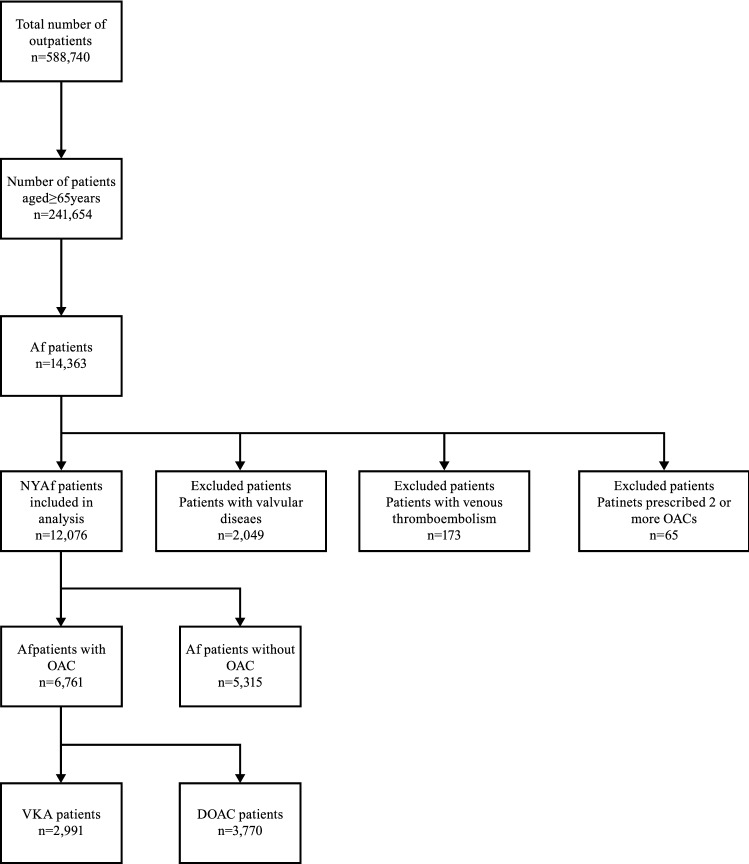
Flowchart of subject extraction (2015). Figure shows extraction proses of patients aged ≥ 65 years from the 2015 outpatient claims data. *Af* Atrial fibrillation, *NVAf* None valvular Atrial fibrillation, *OAC* oral anticoagulant, *VKA* vitamin K antagonist, *DOAC* direct oral anticoagulant.

Subjects were divided into those who were prescribed anticoagulants (n = 6761) and those who were not (n = 5315); the former group was further classified into those who were prescribed VKA (n = 2991) and those who were prescribed DOACs (n = 3770) (Fig. 1). The results from the datasets (2011 to 2014) were obtained using the same procedure.

### Time trends in the numbers of oral anticoagulant prescriptions

**Figure 2:**
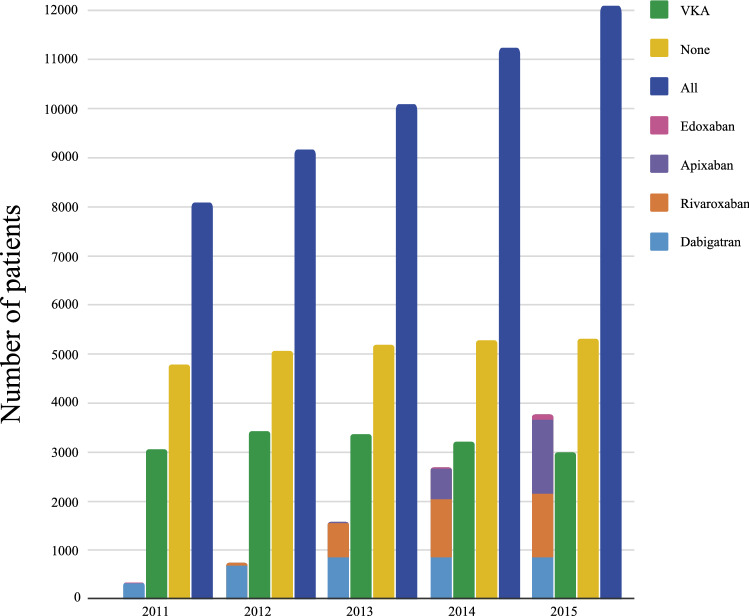
Time trends in the numbers of oral anticoagulant prescriptions. Figure shows time trends in the number of patients who were prescribed VKA, each of the four DOACs, and that of all DOAC-prescribed patients combined, among all. Af patients aged ≥ 65 years. *VKA* vitamin K antagonist, *DOACs* direct oral anticoagulants.

Time trends in the number of patients who were prescribed VKA, each of the four DOACs, and that of all DOAC-prescribed patients combined, among all Af patients aged ≥ 65 years, are shown in Fig. 2. As the total number of Af patients increased from 2011 to 2015, the number of patients who were prescribed DOACs significantly increased (339 to 3770), whereas the number of patients who were prescribed VKA significantly decreased (3051 to 2991).

### Proportions of prescriptions for anticoagulants and AP among all Af patients

**Figure 3:**
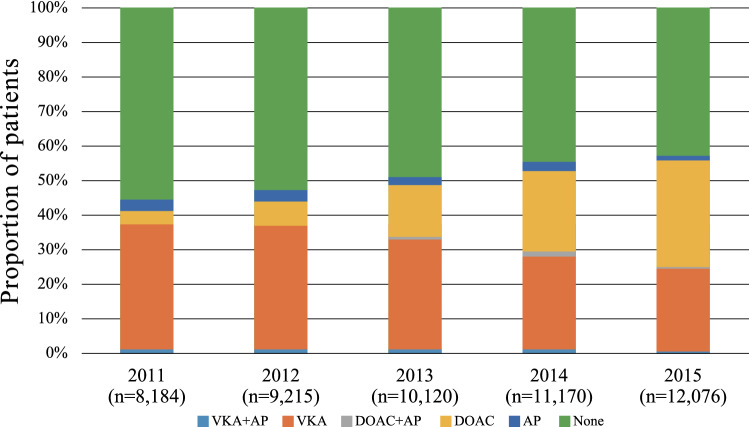
Proportions of prescriptions for anticoagulants and AP among all Af patients. Figure shows time trends in the proportions of prescriptions for anticoagulants and AP among all Af patients aged ≥ 65 years. *VKA* vitamin K antagonist, *DOACs* direct oral anticoagulants.

Time trends in the proportions of prescriptions for anticoagulants and AP among all Af patients aged ≥ 65 years are shown in Fig. 3. The proportion of anticoagulant prescriptions increased from 41 to 56% between 2011 and 2015. The proportion of Af patients who were not prescribed anticoagulants decreased from 56 to 43% between 2011 and 2015. The proportion of AP prescriptions decreased year by year; in 2015, 1% were prescribed AP alone, and 0.8% were prescribed AP and anticoagulants concomitantly.

### Changes over time in the proportions of prescriptions stratified by cerebral infarction risk and bleeding risk

Changes over time in the proportions of anticoagulant prescriptions for Af patients from 2011 to 2015 are shown by group in Table 2. The proportion of anticoagulant prescriptions overall changed from 18% to 26% in Group 1, 37% to 24% in Group 2, 33% to 49% in Group 3, and 54% to 66% in Group 4, with the proportion of DOAC prescriptions reaching 20%, 16%, 29%, and 35% in Groups 1, 2, 3 and 4, respectively, in 2015. In 2015, there were 11,642 Af patients in Groups 3 and 4 combined, and these patients accounted for 96% of all Af patients in that year. In these 11,642 Af patients, the proportion of those who were prescribed anticoagulants was 58% and that of those who were prescribed DOACs was 32%. The proportion of OAC prescriptions was higher in Group 4 than in Group 3.

**Table 2:** The proportion of anticoagulant prescriptions by group of stratified cerebral infarction risk and bleeding risk.

Year	2011							2012							2013							2014							2015						
Group	Af	DOAC		VKA		Total		Af	DOAC		VKA		Total		Af	DOAC		VKA		Total		Af	DOAC		VKA		Total		Af	DOAC		VKA		Total	
	n	n	(%)	n	(%)	n	(%)	n	n	(%)	n	(%)	n	(%)	n	n	(%)	n	(%)	n	(%)	n	n	(%)	n	(%)	n	(%)	n	n	(%)	n	(%)	n	(%)
1	345	8	(2.3)	53	(15)	61	(18)	369	18	(4.9)	57	(15)	75	(20)	398	46	(12)	55	(14)	101	(25)	386	68	(18)	38	(9.8)	106	(28)	397	78	(20)	27	(6.8)	105	(26)
2	19	1	(5.3)	6	(32)	7	(37)	20	1	(5.0)	3	(15)	4	(20)	33	2	(6.1)	9	(27)	11	(33)	26	3	(12)	3	(12)	6	(23)	38	6	(16)	3	(7.9)	9	(24)
Total (1 + 2)	364	9	(2.5)	59	(16)	68	(19)	389	19	(4.9)	60	(15)	79	(20)	431	48	(11)	64	(15)	112	(26)	412	71	(17)	41	(10)	112	(27)	434	84	(19)	30	(6.9)	114	(26)
3	4233	146	(3.4)	1254	(30)	1400	(33)	4768	307	(6.4)	1485	(31)	1792	(38)	5079	718	(14)	1404	(28)	2122	(42)	5547	1279	(23)	1265	(23)	2544	(46)	5897	1706	(29)	1169	(20)	2875	(49)
4	3587	184	(5.1)	1738	(49)	1922	(54)	4058	337	(8.3)	1882	(46)	2219	(55)	4610	798	(17)	1898	(41)	2696	(59)	5211	1340	(26)	1895	(36)	3235	(62)	5745	1980	(35)	1792	(31)	3772	(66)
Total (3 + 4)	7820	330	(4.2)	2992	(38)	3322	(43)	8826	644	(7.3)	3367	(38)	4011	(45)	9689	1516	(16)	3302	(34)	4818	(50)	10,758	2619	(24)	3160	(29)	5779	(54)	11,642	3725	(32)	2991	(26)	6716	(58)

Group 1: CHA_2_DS_2_-VASc = 1, HAS-BLED = 1, 2; Group 2: CHA_2_DS_2_-VASc = 1, HAS-BLED ≧ 3; Group 3: CHA_2_DS_2_-VASc ≧ 2, HAS-BLED = 1,2; Group 4: CHA_2_DS_2_-VASc ≧ 2, HAS-BLED ≧ 3.

*Af Atrial fibrillation.

^†^*VKA* Vitamin K antagonist.

^‡^*DOAC* direct oral anticoagulant.

^§^*CHA*_*2*_*DS*_*2*_*-VASc score* heart failure, hypertension, age ≥ 75 years (doubled), diabetes mellitus, prior cerebral infarction or TIA (doubled), vascular disease, age 65–74 years, female sex.

^||^*HAS-BLED score* hypertension, kidney dysfunction, liver dysfunction, prior cerebral infarction or TIA, prior bleeding or predisposition to bleeding, age ≥ 65 years, use of AP and NSAIDs, alcoholism.

^#^*NSAIDs* non-steroidal anti-inflammatory drugs.

**TIA transient ischemic attack.

## Discussion

These findings from representative NDB data shed light on how anticoagulants are prescribed in Japan. With an increase in DOAC prescriptions, the proportion of anticoagulant prescriptions for Af patients aged ≥ 65 years increased from 41 to 56% from 2011 to 2015. The proportion of anticoagulant prescriptions increased from 33 to 49% in the group of patients with a CHA_2_DS_2_-VASc score ≥ 2 and HAS-BLED scores < 3 (i.e., for whom anticoagulant therapy is recommended), and from 54 to 66% in the group of patients with a CHA_2_DS_2_-VASc score ≥ 2 and HAS-BLED scores ≥ 3, from 2011 to 2015. The number of anticoagulant prescriptions overall increased from 2011 to 2015; the number of DOAC prescriptions surpassed that of VKA prescriptions, which showed a decreasing trend. These results were consistent with previous reports from Denmark, Germany, France, and the United States^3–7^, as well as in Japanese registry studies^8,9^. Clinical studies that examined four DOACs demonstrated no differences between VKA and DOACs^31–36^ and clinical practice guidelines in Japan, and those of other countries now recommend the use of DOACs. This could explain our results as well as those reported previously. Meanwhile, Behdarvand et al. pointed out that intense sales promotion efforts by pharmaceutical companies to market DOACs toward Australian clinicians might have been a driving force behind the increase in DOAC prescriptions^37^. Although there are no such reports in Japan, since DOACs are more expensive than VKA, we cannot deny the possibility that aggressive offer of information provision by companies may have influenced clinicians’ prescribing behaviors.

As for the cause of the increase in the proportion of anticoagulant prescriptions among all Af patients (41% in 2011 to 56% in 2015), it is possible that the introduction of DOACs may have helped reduce the number of patients for whom the attending physician avoided prescribing an anticoagulant. It is conceivable that, when VKA was prescribed as a single therapy, a considerable number of patients were not prescribed anticoagulants. In addition, from 2011 to 2015, there was an increase in the number of Af patients. This suggests that more patients were likely to have been diagnosed with Af, and thus, the number of new patients with Af who were prescribed DOACs also increased. However, the proportion of anticoagulant prescriptions was lower than that reported previously in studies from other countries^3–7^. Given that the risk of developing cerebral infarction is lower among Japanese non-valvular Af patients than among European and American patients^38^, the lower proportion of anticoagulant prescriptions in Japan might reflect a cautious attitude on the part of physicians who prescribe anticoagulant therapy. Moreover, physicians may hold up prescriptions for elderly patients due to a high number of comorbidities and concomitant drugs. Although the efficacy of AP has not been demonstrated^39^, it is still prescribed to some patients with coronary artery disease who have undergone PCI or CABG^40^. However, as concomitant anticoagulants have been reported to be associated with poor prognosis^41^, the use of AP is expected to decrease further in the future.

The proportion of anticoagulant prescriptions, especially DOACs, increased in all four groups. However, this may reflect possible overuse of anticoagulants in Group 1 and 2 patients who have a low need to receive anticoagulant therapy. Moreover, the proportion of anticoagulant prescriptions was higher in Group 4 patients who had a high risk of bleeding compared to Group 3 patients for whom anticoagulant therapy was recommended. This likely resulted from a prescribing behavior that only considers the risk of cerebral infarction when prescribing OAC; in other words, patients with a high HAS-BLED score were administered OAC. Compared with prescription trends in other countries^4^, our results suggest the possibility that anticoagulants may be prescribed based only on cerebral infarction risk. It is also possible that the attitude of clinicians may change in the direction of “if in doubt, prescribe.” With the introduction of anticoagulant therapy, there has emerged a need to consider safety and reduce risks. Efforts should be made to control blood pressure to prevent bleeding, to instruct patients to reduce alcohol consumption, and to avoid the use of NSAIDs and other drugs^42^. Whether the increase in DOAC administration reduced the risk of developing cerebral infarction or increased the risk of bleeding in Af patients should be evaluated in future studies.

This study has several limitations. First, we could not use whole the claims database of NDB, because of the NDB sampling dataset comprised single-month data and not a continuous set of data. Accordingly, whether patients were newly prescribed DOACs or switched from VKA to DOACs could not be verified. Similarly, at the time of OAC prescription, whether it was prescribed in consideration of CHA_2_DS_2_-VASc and HAS-BLED scores could not be verified. Second, since insurance claims data contain no descriptions of laboratory test values (INR, kidney function, blood pressure, body weight, etc.), CHA_2_DS_2_-VASc and HAS-BLED scores were calculated based on disease names and drugs used. This likely resulted in higher scores relative to actual risks, given that patients with hypertension were identified based on the presence of administered anti-hypertensive drugs, that is, some patients who were included in this patient group might not have had hypertension. For example, there might have been cases in which patients were assigned a disease code for hypertension so that antihypertensive drugs could be administered for the purpose of treating IgA nephropathy. Even though such cases might not be common, this could have led to high CHA_2_DS_2_-VASc and HAS-BLED scores. Finally, comorbidities and concomitant drugs were also likely confounders, but were not fully assessed in this analysis.

## Conclusion

From 2011 to 2015, anticoagulant prescriptions for patients with non-valvular Af rapidly shifted from VKA to DOACs, and the proportion of total anticoagulant prescriptions increased in Japan. While the proportion of anticoagulant prescriptions for patients with a high risk of cerebral infarction increased, that for patients at a high risk of bleeding also increased. Due to the widespread use of DOACs, the profiles of these two risks, which are associated with anticoagulant prescriptions in non-valvular Af patients, are drastically changing.

## Supplementary Information

Supplementary Table S1.

## Data Availability

The datasets used and/or analyzed during the current study are available from the corresponding author on reasonable request.

## Abbreviations

Af: Atrial fibrillation
VKA: vitamin K antagonist
DOACs: direct oral anticoagulants
NDB: the National Database of Health Insurance Claims and Specific Health Checkups of Japan
ICD-10: the 10th revision of the International Classification of Diseases
ATC: the Anatomical Therapeutic Chemical Classification System.
OACs: oral anticoagulants
AP: antiplatelet agent
NSAIDs: non-steroidal anti-inflammatory drugs
CHA_2_DS_2_-VASc score: heart failure, hypertension, age ≥ 75 years (doubled), diabetes mellitus, prior cerebral infarction or TIA (doubled), vascular disease, age 65–74 years, female sex
HAS-BLED score: hypertension, kidney dysfunction, liver dysfunction, prior serebral infarction or TIA, prior bleeding or predisposition to bleeding, age ≥ 65 years, use of AP and NSAIDs, alcoholism
TIA: transient ischemic attack
PCI: percutaneous coronary intervention
CABG: coronary artery bypass grafting
