# Supplementary material for: Trend of anticoagulant therapy in elderly patients with atrial fibrillation considering risks of cerebral infarction and bleeding
Source: Sci Rep. 2023 Jan 5;13:192. doi: 10.1038/s41598-022-26741-7 (PMC9814101; doi:10.1038/s41598-022-26741-7)
Supplement: Supplementary file 1 — Supplementary Table S1. [file 41598_2022_26741_MOESM1_ESM.pdf]

Trend of anticoagulant therapy in elderly patients with atrial fibrillation considering risks of cerebral infarction and bleeding

Noriko Tsuji [a], MPH, Yoshimitsu TAKAHASHI [a], PhD, Michi Sakai [b], PhD, Shosuke Ohtera [c], PhD, Junji Kaneyama [d], MD, MPH, Kosai Cho [e], MD, PhD, Genta KATO [f], MD, PhD, Shigeru Ohtsuru [e], MD, PhD, Takeo Nakayama [a], MD, PhD

[a] Department of Health Informatics, Graduate School of Medicine and Public Health, Kyoto University, Kyoto, Japan; [b] Comprehensive Unit for Health Economic Evidence Review and Decision Support, Ritsumeikan University, Kyoto, Japan; [c] Center for Outcomes Research and Economic Evaluation for Health, National Institute of Public Health, Wako, Japan; [d] Akita Cerebrospinal and Cardiovascular Center, Second Department of Cardiology, Akita, Japan; [e] Department of Primary Care and Emergency Medicine, Kyoto University Hospital, Kyoto Japan; [f] Solutions Center for Health Insurance Claims Integrated Clinical Education Center Kyoto University Hospital, Kyoto, Japan.

Supplementary Table S1. Diagnoses, surgical procedures, and pharmacotherapy used for defining the population and comorbidity

Non-valvular atrial fibrillation

Presence of	*ICD10*: I48
Absence of	*ICD10*: I05, I06, I34, I35

Comorbidity

Alcohol abuse	Defined from diagnosis	*ICD10*: E244, E52, G312, G621, G721, I426, K292, K701-704, 709, K860, T51
Bleeding history	Defined from diagnoses of gastrointestinal, intracranial, urinary tract, or airway bleeding	*ICD10*: I60-I62, I690, J942, K25-28, K920-K922, N029, R04, R31, S064-S066
Chronic kidney disease	Defined from diagnosis	*ICD10*: I120, N02-N08, N11-N12, N14, N18-N19, N26, Q610-Q615, Q618-619,

Diabetes mellitus	Defined from treatment	*Treatment*: Glucose-lowering medication
Drug use	Defined from treatment	*Treatment*: Non-steroidal anti-inflammatory drugs or platelet inhibitors
Heart failure	Defined from diagnosis	*ICD10*: I50
Hypertension	Defined from combination of diagnosis and treatment with antihypertensive drugs	*ICD10: I10,I11,I12,I13,I15*<br>*Treatment*: Adrenergic α-antagonist, non-loop-diuretics, vasodilators, beta blockers, calcium channel blockers, and renin-angiotensin system inhibitors
Ischemic heart disease	Defined from diagnosis	*ICD10*: I20-I25
Liver disease	Defined from diagnoses of liver cancer, chronic liver	*ICD10*: B15-B19, C22, D684, K70-K76, A188, A527, B008, B251, B268, B338, B581, B661

	disease, liver surgery, cirrhosis, and hepatitis	B659, B675, B670, B678, B89 D868, E106, E116, E146, E740 E888, Z944
Myocardial infarction	Defined from diagnosis	*ICD10*: I21-I22
Peripheral artery disease	Defined from diagnosis	*ICD10*: I700, I702-I709
Stroke or systemic thromboembolism history	Defined from diagnoses of peripheral artery embolism, stroke, and transient ischaemic attack	*ICD10*: G458-G459, I63-I64,I69, I74
Vascular disease	Defined from diagnoses of myocardial infarction, peripheral artery disease, and aortic plaque	*ICD10*: I21-I22, I700, I702-I709

ICD10: 10th revision of the International Classification of Diseases system
